# Evaluation of a fast method of EPID‐based dosimetry for intensity‐modulated radiation therapy

**DOI:** 10.1120/jacmp.v11i2.3185

**Published:** 2010-04-19

**Authors:** Benjamin E. Nelms, Karl H. Rasmussen, Wolfgang A. Tomé

**Affiliations:** ^1^ Department of Human Oncology University of Wisconsin, School of Medicine and Public Health 600 Highland Ave. Madison WI 53792 USA; ^2^ Department of Medical Physics University of Wisconsin, School of Medicine and Public Health 600 Highland Ave. Madison WI 53792 USA; ^3^ Canis Lupus LLC Sauk County WI 53561 USA

**Keywords:** intensity‐modulated radiation treatment, EPID, portal dosimetry, quality assurance

## Abstract

Electronic portal imaging devices (EPIDs) could potentially be useful for intensity‐modulated radiation therapy (IMRT) QA. The data density, high resolution, large active area, and efficiency of the MV EPID make it an attractive option. However, EPIDs were designed as imaging devices, not dosimeters, and as a result they do not inherently measure dose in tissue equivalent media. EPIDose (Sun Nuclear, Melbourne, FL) is a tool designed for the use of EPIDs in IMRT QA that uses raw MV EPID images (no additional build‐up and independent of gantry angle, but with dark and flood field corrections applied) to estimate absolute dose planes normal to the beam axis in a homogeneous media (i.e. similar to conventional IMRT QA methods). However, because of the inherent challenges of the EPID‐based dosimetry, validating and commissioning such a system must be done very carefully, by exploring the range of use cases and using well‐proven “standards” for comparison. In this work, a multi‐institutional study was performed to verify accurate EPID image to dose plane conversion over a variety of conditions. Converted EPID images were compared to 2D diode array absolute dose measurements for 188 fields from 28 clinical IMRT treatment plans. These plans were generated using a number of commercially available treatment planning systems (TPS) covering various treatment sites including prostate, head and neck, brain, and lung. The data included three beam energies (6, 10, and 15 MV) and both step‐and‐shoot and dynamic MLC fields. Out of 26,207 points of comparison over 188 fields analyzed, the average overall field pass rate was 99.7% when 3 mm/3% DTA criteria were used (range 94.0–100 per field). The pass rates for more stringent criteria were 97.8% for 2 mm/2% DTA (range 82.0–100 per field), and 84.6% for 1 mm/1% DTA (range 54.7–100 per field). Individual patient‐specific sites as well, as different beam energies, followed similar trends to the overall pass rates.

PACS number: 87.53.Dq; 87.66.Jj

Conflict of Interest Statement: Canis Lupus LLC performs consulting for Sun Nuclear Corporation.

## I. INTRODUCTION

Modern radiation therapy is personalized to the specific patient and the specific treatment situation. Therefore, resulting treatment field arrangements and treatment fields are inherently more complex. Such personalized therapy requires rigorous commissioning of delivery systems and treatment planning systems (TPS). It also requires that the medical physicist conduct per‐plan/per‐patient quality assurance to ensure the prescribed treatment dose is accurate and achieved in preset and clinically acceptable error tolerances. Therefore, intensity‐modulated radiation therapy (IMRT) QA is performed on a plan‐specific and, in most cases, on a per‐treatment‐field basis.

One general IMRT QA method that is essential to commissioning and common in per‐plan QA (for nonrotational plans) is to treat IMRT fields one by one onto a measurement phantom, measuring a dose plane normal to the beam direction and comparing to a TPS‐calculated dose plane. Such a method verifies dose across the modulated 2D profile of each beam and enables the clinical physicist to: 1) assess the ability of the TPS to calculate dose given complex beam modulation, 2) assess that the treatment delivery system is capable of delivering the complex fields, and 3) diagnose and troubleshoot failures of the TPS and/or treatment delivery system. The complex dose gradients present in IMRT treatment fields require that these verification measurements have both high data density (i.e. measurement points per unit area) and high spatial resolution (i.e. small detectors) to test the entirety of the optimized field. The use of small detectors in measurements is essential to avoid the problem of volume averaging that is present when using large detectors.

However, while thoroughness is vital to IMRT QA, it is coupled with the practical need for efficiency. During the last few years, IMRT has become the standard of care for many treatment sites due to its ability to better conform the dose to the treatment target and allow the planner to avoid critical normal structures as compared to 3D conformal therapy.^(^ ^1^ ^–^ ^3^ ^)^ The increasing number of patients being treated using IMRT necessitates the use of an efficient patient‐specific QA process to allow for completion of the process in a timely manner.

Current IMRT QA methods using film, 2D ion chamber arrays, or 2D diode arrays have inherent limitations. Film provides data at a high resolution. However, doing QA with film is a time‐consuming process due to phantom setup, film development (including time‐dependent chemical reactions that affect both traditional and radiochromic film), and film analysis. Furthermore, there are issues with uncertainties due to processor artifacts and, with the advent of electronic records, film processors may soon cease to be standard equipment in radiotherapy departments. Ion chamber and diode arrays allow for fast analysis; however, they both have a reduced data density compared to film. Additionally, ion chambers exhibit marked volume averaging that requires the TPS‐calculated dose to be blurred prior to IMRT QA.^(4)^

Because of their online efficiency and data density, portal imagers/MV EPIDs have received attention as potential IMRT QA devices.^(^ ^5^ ^–^ ^7^ ^)^ EPIDs are typically standard on modern linear medical accelarators. Their primary use was for patient localization via portal imaging and thus they were designed to exhibit high contrast and spatial resolution. However, a secondary use of EPIDs in absolute dose‐based, patient‐specific IMRT QA (if proven accurate) could be extremely useful since it would shorten and streamline the patient‐specific IMRT QA process.

With an EPID, an image is acquired via the generation of electrons in a copper plate by an incident MV photon beam. These electrons then interact with a scintillating material. The visible light generated in the scintillator then interacts with a flat panel photo‐diode for each point on the array, and the generated charge is recorded over a 2D grid. The EPID itself is “online” on the Linac, meaning that it is housed in a retractable arm that can be positioned automatically in the beam path without any manual setup in a highly reproducible manner. For positioning accuracy, there will be no need to perform additional QA tests for IMRT QA beyond those that are necessary to assure positioning accuracy of the EPID for MV portal imaging. Typical EPID data resolution (pixel size), data density (pixels per area), and array size are shown in Table 1.

**Table 1:** Summary of commercially available EPID units used in this study.

*Model type*	*Active area (pixels)*	*Spatial resolution (mm/pixel)*
Varian aS500	512×384	0.784
Varian aS1000	1024×768	0.392
Siemens OptiVue 500	512×512	0.4
Siemens OptiVue 1000	1024×1024	0.4

However, with these potential benefits of high data density and high resolution for EPID‐based IMRT QA, there are also inherent problems associated with EPID‐based dosimetry. The raw EPID image is not a dose image, and the EPID response deviates from what would be expected based on water‐based dose measurements and cannot simply be “calibrated” to get from image‐to‐dose. EPID shows different response with respect to head scatter,^(6)^ spectral changes in MLC transmission regions,^(6)^ ^,^ ^(8)^ and for scatter characteristics given the material makeup of an EPID compared to tissue.

In this work, we investigate the accuracy of a newly developed method of EPID‐based dosimetry (EPIDose, Sun Nuclear Corporation, Melbourne, FL) that attempts to solve many of the problems inherent in EPID‐based dosimetry via an EPID image‐to‐dose plane conversion algorithm. It is imperative that testing the accuracy of an EPID‐based solution be done by making absolute dose comparison vs. trusted standard IMRT QA measurement systems, and not by only comparing to TPS calculations. To benchmark new QA measurement methods by only comparing to TPS calculations, as has been done previously,^(9)^ would be using an incorrect standard, if the measurement/QA system's intention is largely to assess the TPS's ability to calculate complicated dose distributions. Of course, if it were known that the TPS calculations are perfect, then using it as the standard would be valid; however, if the TPS calculation were perfect, we would have much less of a need for IMRT QA systems in the first place. Therefore, assessing the accuracy of a new measurement strategy is best done by comparing against an already proven measurement method.^(10)^ ^,^ ^(11)^

In this work, we compare IMRT QA absolute dose planes acquired with EPIDose to measurements acquired via a well‐established 2D IMRT QA dose measurement array, MapCHECK (Sun Nuclear, Melbourne, FL), which has been discussed in the literature.^(^ ^10^ ^,^ ^12^ ^–^ ^14^ ^)^ It should be noted that, due to the inherent challenges of the EPID‐based dosimetry already mentioned, validating and commissioning such as system must be done very thoroughly and carefully over a wide range of potential clinical conditions.

The goal of this study was to determine if an EPID‐to‐dose commercial conversion algorithm is accurate at predicting absolute dose planes for IMRT QA over a wide variety of conditions (beam energy, beam modulation, field sizes, and Linac/EPID models).

## II. MATERIALS AND METHODS

### A. IMRT plan details

Twenty eight plans were acquired from previously planned IMRT treatments encompassing 188 fields. Treatment field sites included prostate (n=142), brain/head and neck (n=29), and lung (n=17). IMRT fields were planned using the following beam energies: 6 MV (n=141), 10 MV (n=42), or 15 MV (n=5) from standard linear accelerators: (Varian (n=167) and Siemens (n=21)). Two head and neck plans used a composite split field analysis through the overlaying of separate measurements; only the composite fields (i.e. sum of split fields) were considered in these cases. Both dynamic (sliding window) (n=33) and step‐and‐shoot (n=155) IMRT fields were analyzed.

Six centers submitted anonymous patient plans, 2D diode array measurements, and EPID images for this study. Depending on the center, plans were created on either ${\text{Pinnacle}}^{3}$ (Philips Radiation Oncology Systems, Fitchburg, WI) (n=16), XiO (CMS, St. Louis, MO) (n=7), or Eclipse (Varian Medical Systems, Palo Alto, CA) (n=5). Planar dose images were generated using the respective TPS at a 5 cm depth and distance of either 100 or 105 source‐to‐plane distance (SPD). Additionally, multileaf collimator (MLC) files or DICOM‐RT plans were exported for all patients, as these are required by the EPIDose algorithm.

In addition to clinical IMRT fields, some simple test fields were created and analyzed as well, allowing for controlled probing/diagnosis of sources of possible weaknesses in the EPIDose methodology. Seven test fields were designed: six small MLC‐shaped fields (ranging from 1×1 to 7×7) inside a fixed 10×10 primary collimation (Varian linear accelerator model), and a single “inverted dose pyramid” step‐and‐shoot IMRT field consisting of 11 MLC segments.

### B. Acquisition method

**Figure 1:**
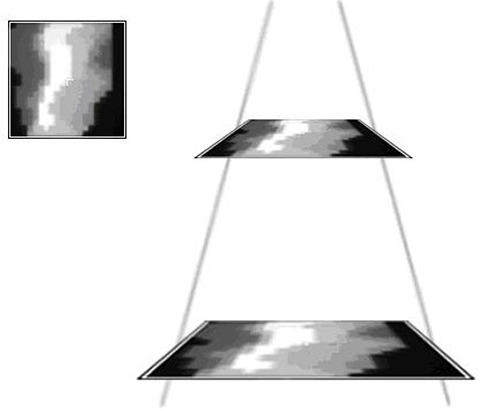
In the first conversion step, the EPID image is geometrically back‐projected from the EPID dose plane distance to the desired dose plane distance (e.g., 140 SDD to 100 SPD).

**Figure 2:**
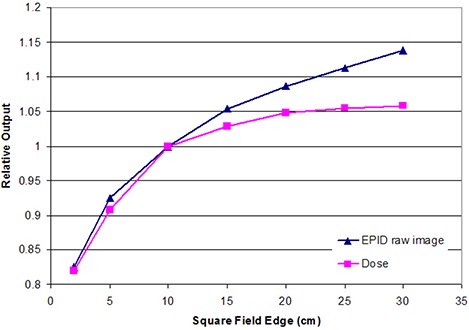
Field size response of one representative 6X EPID. In the second conversion step, the field size response variation is accounted for through software analysis of all MLC segments. The EPID image is then corrected for each exposed section and relative contribution of control points.

**Figure 3:**
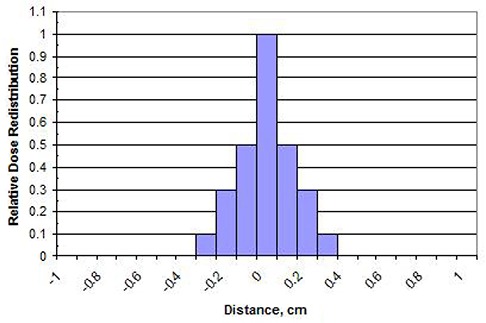
In the third step, the pixel values in the EPID image are convolved with a redistribution kernel. This creates a water equivalent electron dose distribution (for the modeled QA plane depth, in phantom). Redistribution is very dependent on beam energy and the simulated QA depth‐in‐phantom, and to a certain extent on the EPID image acquisition and processing employed by the user.

**Figure 4:**
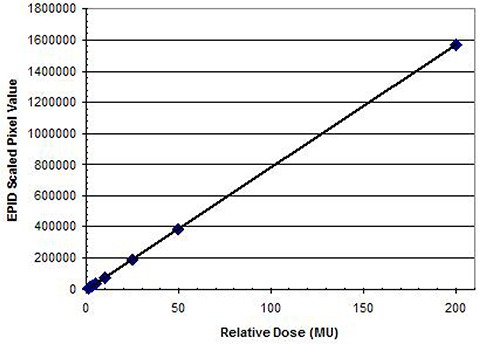
In the fourth conversion step, a 2D wide field calibration map is applied to correlate relative individual pixel values to absolute dose points.

Images were acquired using either AM Maintenance, VARIS, or Aria for plans delivered using a Varian linear accelerator and beam view coherence when employing a Siemens linear accelerator. A dose rate between 300–400 MU/min was used and EPID images were acquired below the saturation effect limit.^(6)^ The source‐to‐detector distance (SDD) varied between treatment centers (range 120–145 cm), but was consistent for all fields delivered by each center. Modeling data was acquired to account for machine‐specific characteristics. No additional buildup material was directly placed on the EPID, which eliminates the possibility of sag in the EPID arm due to the weight of buildup material placed directly on the EPID front face. In order to estimate the variation in EPID response due to head scatter/field size, MLC defined field sizes of 1 ×1 cm, 2×2 cm, 5×5 cm, 10×10 cm, 15×15 cm, 20×20 cm, 25×25 cm, and 30×30 cm were measured for 100 MU at the appropriate SDDs at each treatment facility (for both dose and EPID relative response). A correction ratio for MLC transmission regions (for each subsegment of an IMRT field) is also allowed in the generated model based on these measurements. A “dose redistribution kernel” was generated and commissioned for each model in order to convolve raw EPID response to dose‐equivalent scatter at the desired QA depth in phantom. Finally, to generate a wide field calibration to absolute dose, a 20×20 cm field size was also measured with 25, 50, 100, and 200 MU (again, for both dose and raw EPID response). Typical physics model data acquired and generated for this study for each employed EPIDose model is illustrated in Figs. 1–4. A detailed explanation of the conversion of a raw EPID image to an absolute planar dose image is provided in the following section (C. EPID image conversion).

A total number of 188 fields were measured and analyzed. It should be noted that each permutation of Linac, geometry setup (EPID and dose plane), and beam energy requires its own EPIDose model. Therefore, all plan IMRT fields were measured using the same setup conditions as those used in generation of the model for each center (i.e. each center used independent and customized model parameters, determined by their equipment and setup preferences).

### C. EPID image conversion

The pixels of an EPID image do not scale directly to planar dose. Used in their raw form, EPID images may be useful to qualitatively assess beam modulation and perhaps verify a beam's pattern, scale, and orientation. However, in this raw form, they are not useful in assessing the delivered absolute dose vs. the planned absolute dose. Without an accurate and robust method to “convert” an EPID image into an absolute dose plane (analogous to what a 2D dose array or film placed in a flat phantom normal to the beam might provide), the potential utility of EPID images in IMRT QA is severely limited.

**Figure 5:**
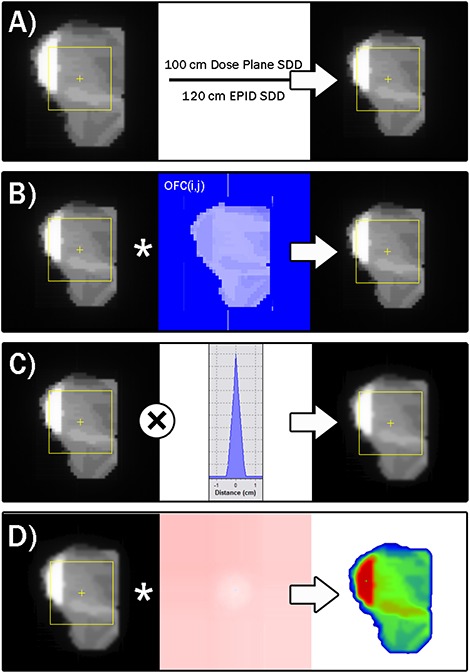
Progression of conversion of a raw EPID image into an absolute dose plane. Panel A illustrates the geometric scaling from the EPID distance to the desired dose plane distance. Panel B shows the scaling of each pixel by the 2D output correction map, derived using the MLC segment data (size, shape, relative meterset weight). Panel C shows the convolution with the dose redistribution kernel to emulate dose distribution in a water phantom at the modeled QA dose plane depth. Panel D illustrates the conversion to absolute dose values using the wide field calibration map.

To convert an EPID image to a virtual dose plane in water, a prototype of a commercial system was employed – MapCHECK in conjunction with the EPIDose module version 4.00.01 (Sun Nuclear, Melbourne, FL). The process used by this system is demonstrated in Figs. 1–4. All calculations were performed on a 1.8 GHz Intel Dual core computer with a Windows XP operating system. The details of the algorithm were in “patent pending” status at the time of writing this article; however, the vendor agreed to share the algorithm mechanisms for publication in this article. The processes carried out by the EPIDose algorithm are specified in the four serial mechanisms described below. (An actual illustration of the intermediate results of each mechanism for an actual EPID image‐to‐dose plane conversion is given in Fig. 5.)

1. It is not required by the conversion algorithm that the EPID panel geometry match the desired dose plane geometry. That is, if a physicist wants to acquire EPID images at a distance from the source of X but desires the simulated absolute dose plane to be at distance Y (to match his/her protocol for per‐beam QA), the algorithm allows this. Therefore, the first step of the conversion mechanism is a simple geometric back‐projection technique applied to the raw EPID image to scale its pixels to the desired source‐to‐dose plane distance (see Panel A of Fig. 5). This accounts for the difference in beam divergence from the measured EPID SDD to the TPS generated dose plane distance (and in the case of this paper, to the verifying dose plane measured by the diode array).
  Table 2 summarizes the varying source‐to‐EPID distances and source‐to‐QA dose plane distances preferred by the institutions contributing to this study. The preferred EPID SDDs ranged from 100 cm to 140 cm. As for the QA dose planes, the preferred SDDs were 100 cm at five of the six institutions, and 95 cm for the other institution.
2. The second mechanism of the conversion algorithm is to apply a weighted 2D correction map to the scaled EPID image resulting from the previous step. This correction map is required in order to account for two important truths about EPID images: 1) the EPID response vs. the field shape and location varies differently than does the dose response measured in the QA phantom (i.e. the EPID pixel response requires a different head scatter/source model correction than that which models absolute dose); and 2) the EPID response in the low dose rate regions blocked by MLC leaves can be significantly different than the absolute dose response in those same regions.
  To build the correction map, the algorithm must know the segment parameters and relative meterset weights, and therefore the MLC data must be imported (either as a DICOM RT Plan or a MLC file) into the EPIDose software in the step prior to EPID image‐to‐dose plane conversion. The parameters that drive the output factor corrections per segment are derived from the relative output vs. field size curves required in the modeling parameters. The parameter that drives the corrections in each segment's MLC transmission region is a single value called the “Dose/EPID for MLC Transmission.”
  For each IMRT segment (which can be just a few for some SMLC beams, or hundreds in the case of DMLC, RapidArc, or VMAT beams), the whole field correction map is updated for each location in the beam's eye view (BEV) (i.e. each pixel in the newly scaled 2D array). The corrections per‐pixel, per‐segment are equal to either: a) for exposed regions in the BEV, the estimated Dose/EPID response as a function of integrating the open MLC region over the dose response and dividing by the integration over the EPID response, or b) for MLC transmission regions, the single Dose/EPID correction factor. Each pixel's correction factor for each segment is weighted by the estimated relative dose contribution of that segment to the whole field, which is derived from each segment's portion of the total monitor units (the MLC transmission regions' contributions being modified further by the MLC transmission).
  The cumulative 2D output factor correction (OFC) map for IMRT field “F” can be summarized by the following equation:
  (1) $$OFC_{F}(i,j)=\sum_{S=1}^{N}CD_{S}(i,j)\times \left[\frac{\Delta Meterset_{S}}{Cumulative\;Meterset_{F}}\right]$$
  Where N=total of number MLC segments per IMRT field, (i,j) is the location in the 2D BEV output factor correction matrix, ${\text{CF}}_{S}\left(i,j\right)=\text{estimated}$ ${\left(\text{Dose}\;\text{output}/\text{EPID}\;\text{output}\right)}_{S}$ if (i,j) is in open region, and ${\text{CF}}_{s}\left(i,j\right)={\left(\text{Dose}/\text{EPID}\right)}_{\text{MLC}\;\text{Transmission}}$ if (i,j) is under MLC leaves.
  The accumulation of pixel‐by‐pixel corrections over all segments results in a normalized 2D array correction map that is multiplied by the geometrically scaled EPID image to give an output‐corrected 2D array of values (see Panel B of Fig. 5).
3. After the 2D exposure region normal to the IMRT field has been corrected for relative output differences between the EPID and absolute dose responses, a correction is applied to redistribute the EPID response to emulate what would have occurred if absolute dose had been measured in a homogeneous phantom at the QA dose plane depth.
  The EPID image‐to‐dose algorithm tested here does not require any buildup material to be placed directly on the EPID panel. The scatter conditions of an EPID panel (with material makeup much different than a water phantom) are much different than that of dose‐to‐tissue/water at normal QA plane depths. The dose‐at‐depth is qualitatively “more scattered” and consistently so at any given depth and, therefore, a “dose redistribution kernel” as described earlier is convolved with the scaled/output‐corrected 2D pixel array to simulate the broader dose deposition spread in water relative to the higher density EPID (see Panel C of Fig. 5).
  The kernels used by each of the participating institutions was a function of the modeled dose depth and energy, and was determined by a manual fitting by each institution and implemented without further modification (i.e. there was no attempt by the authors to optimize the dose redistribution kernels).
4. The final step in converting the 2D EPID image to a simulated 2D absolute dose array at depth in a homogeneous phantom is to calibrate each pixel to absolute dose (see Panel D of Fig. 5). To achieve this, a stored 2D wide field calibration map is applied to the scaled/output‐corrected/kernel‐convolved 2D array of pixels. A wide field calibration map is useful because there are off‐axis differences for EPID panels usually resulting in raw EPID images for large rectangular fields that are much flatter in response than a dose profile measured at depth.
  For large, open fields, the off‐axis response can vary due to flood field interactions, but the algorithm analyzed in this paper employs a single wide field calibration map. (For this reason, we made sure to include a variety of IMRT field sizes to test both large and small fields.) The calibration map is generated in the physics modeling process by irradiating a series of wide fields (in our case, we used 20×20 fields) at varying monitor unit levels (25, 50, 100, and 200 MU) to both the EPID and the diode array.

**Table 2:** Summary of the geometry parameters used for the six participating sites. The QA dose plane parameters were the ones used for both a) actual MapCHECK measurements (for comparisons/commissioning), and b) simulated dose planes resulting from a conversion of the EPID images.

*Site / EPID Vendor*	*Source‐to‐EPID Distance (cm)*	*Source‐to‐QA Dose Plane (cm)*	*QA Dose Plane Depth (cm)*
A / Varian	140.0	100.0	5.0
B / Varian	100.0	100.0	10.0
C / Siemens	100.0	100.0	5.0
D / Varian	120.0	100.0	5.0
E / Varian	105.0	95.0	5.0
F / Varian	105.0	100.0	5.0

The analysis software follows the scaling, output correction, and dose redistribution steps for these large EPID calibration images as outlined above, and then creates a pixel‐by‐pixel lookup table of pixel value‐to‐dose calibration factors by referencing against the measured calibration absolute dose planes. The resulting calibration map is stored with the physics model. For large IMRT fields that fall outside the calibrated region, the closest geometric calibration factor is used, so it is important to analyze very large fields with scrutiny. (We have learned that wider calibration fields are now available, but in this study we used 20 cm×20 cm fields).

Note that the calibration map has to be reassessed on a periodic basis to detect any drifts, and it must be checked and, if necessary, recreated if any software parameters of the EPID are changed or after servicing of the EPID. However, in most cases we found there was very little drift in between servicing activities.

### D. Diode array measurements and comparison

A 2D diode array (MapCHECK, Sun Nuclear, Melbourne, FL) was used to measure the same MLC defined field sizes at the specified IMRT QA setup (detector distance, detector depth) for each EPIDose model. The diode array was calibrated as specified in the user's manual and was chosen due to the similar resolution of EPID imaging (0.784 mm) and the diode array (0.8 mm).^(6)^ ^,^ ^(10)^ The accuracy and reproducibility of 2D diode arrays has been previously demonstrated in several studies.^(10)^ ^,^ ^(12)^

After conversion of the raw EPID image, the simulated dose planes were compared to the diode array measured dose using the Van Dyk percent difference setting (global dose normalization, not local) and distance‐to‐agreement (DTA) method.^(10)^ ^,^ ^(15)^ All dose analysis was done on absolute dose, not relative dose. DTA was used because it is more stringent than the Gamma Index. A lower dose threshold for analysis was set at 10%, so that low dose values would not artificially increase pass rates (given the usage of global dose normalization, a.k.a. “Van Dyk” percent difference).

The analysis software allowed for a shift to be applied in 2D coordinates. Applying a shift to datasets corrects for setup (or systemic detector) misalignment, however it also could give a user the ability to search for an incorrectly high agreement for individual fields. That is, a shift error could, in theory, be the result of a TPS or delivery error, and masking it by moving data around for a best fit might be the wrong thing to do. In this study, EPID converted dose planes and 2D diode array measurements were considered to be fixed relative to each other for all measurements taken on the same day from an individual center. Therefore, all plans were analyzed with a single uniform alignment shift from each center.

It is worth restating that the comparisons performed in this study are of the true commissioning variety; that is, we compared the proposed measurement technique (EPIDose) vs. a proven and trusted measurement technique (MapCHECK). Some previous studies analyzing proposed measurement/QA systems have used comparisons with the TPS calculations as the method of analyzing/proving the proposed system.^(9)^ ^,^ ^(16)^ We believe this strategy, though certainly of value, is not sufficient when one is attempting to analyze the efficacy of a new QA system.

## III. RESULTS

In this study, 188 IMRT treatment fields from 28 distinct IMRT plans were analyzed. Table 3 shows the cumulative results of comparing planar doses measured with the commercial 2D diode array and the converted EPID‐to‐dose planes for the 188 IMRT beams analyzed. The average overall field pass rate was 99.7%±0.1% when the 3 mm/3% DTA/Percent Dose Agreement combination criteria were used. If the DTA/Percent constraints were tightened, the pass rate decreased slightly to 97.8%±0.4% for 2 mm/2%. When the constraints were tightened even further to 1 mm/1%, the pass rate dropped to 84.6%±1.3%.

**Table 3:** Percent difference/DTA comparison between EPID converted dose planes to 2D diode array measurements for three analysis criteria (% difference/DTA): 3 mm/3%, 2 mm/2%, and 1 mm/1%. The averages include the 95% statistical confidence intervals.

*Field Type*	3 mm/3% *Pass Rate*		2 mm/2% *Pass Rate*		1 mm/1% *Pass Rate*		*# of Plans*	*# of Fields*
	*Average*	*Range*	*Average*	*Range*	*Average*	*Range*		
**All Fields**	99.7±0.1	94.0–100	97.8±0.4	82.0–100	84.6±1.3	54.7–100	28	188
**Prostate**	99.8±0.1	97.4–100	98.3±0.4	82.0–100	86.4±1.4	54.7–100	21	142
**Lung**	99.7±0.3	97.8–100	97±1.2	92.6–100	81±4.1	60.6–95.2	2	17
**Brain/Head & Neck**	99.2±0.4	94.0–100	96±1.2	87.6–100	78.3±2.6	62.5–92.3	5	29
**High Energy**	99.9±0.1	97.4–100	98±1.0	82.0–100	84.5±3.3	54.7–96.0	7	47
**Low Energy**	99.7±0.1	94.0–100	97.8±0.4	87.6–100	84.7±1.3	60.6–100	21	141

**Figure 6:**
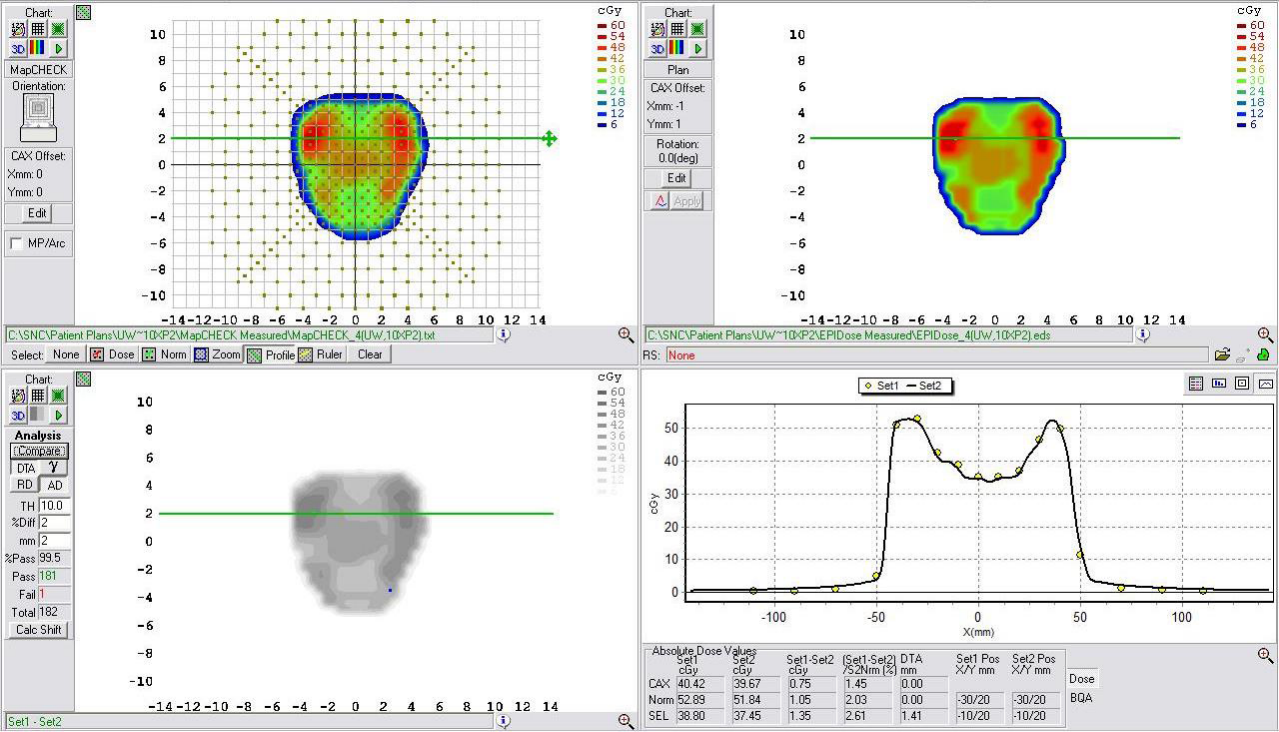
Example comparison of a converted EPID dose plane to a 2D diode array dose distribution for a typical prostate field using a 2 mm/2% DTA comparison.

**Figure 7:**
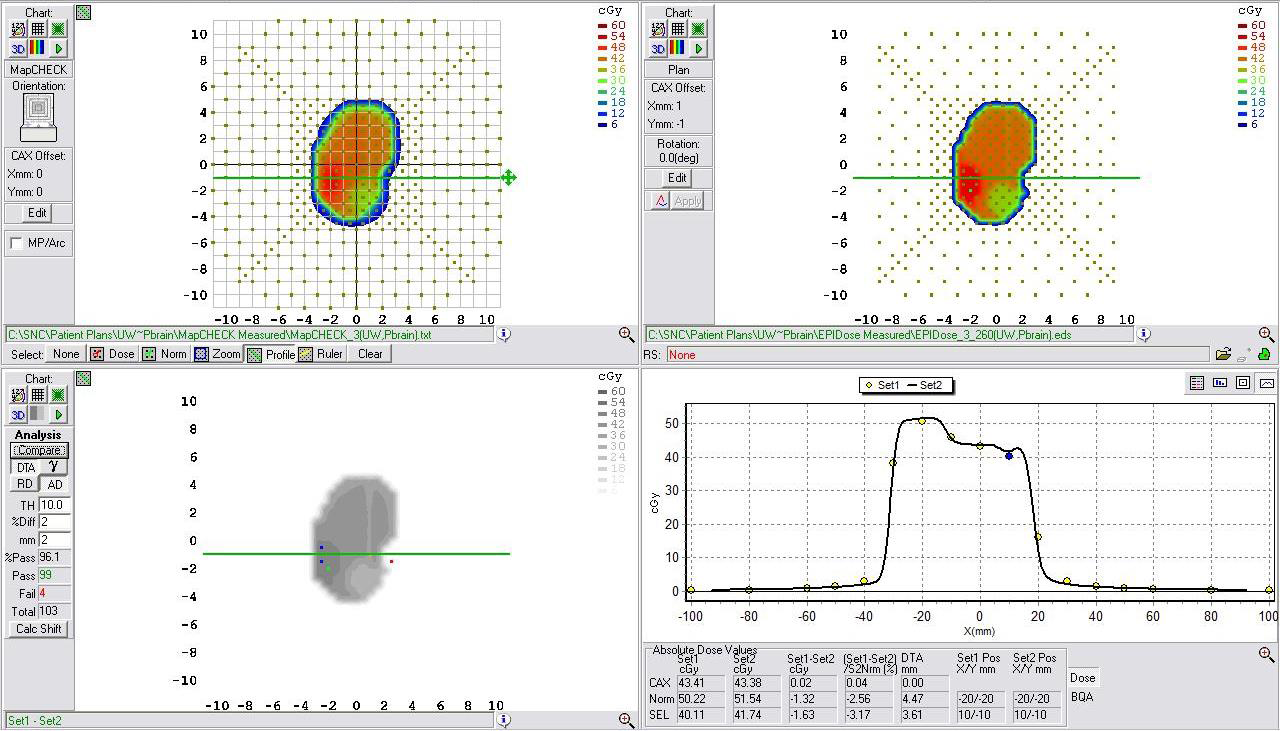
Example comparison of a converted EPID dose plane to a 2D diode array dose distribution for a typical brain field using a 2 mm/2% DTA comparison.

**Figure 8:**
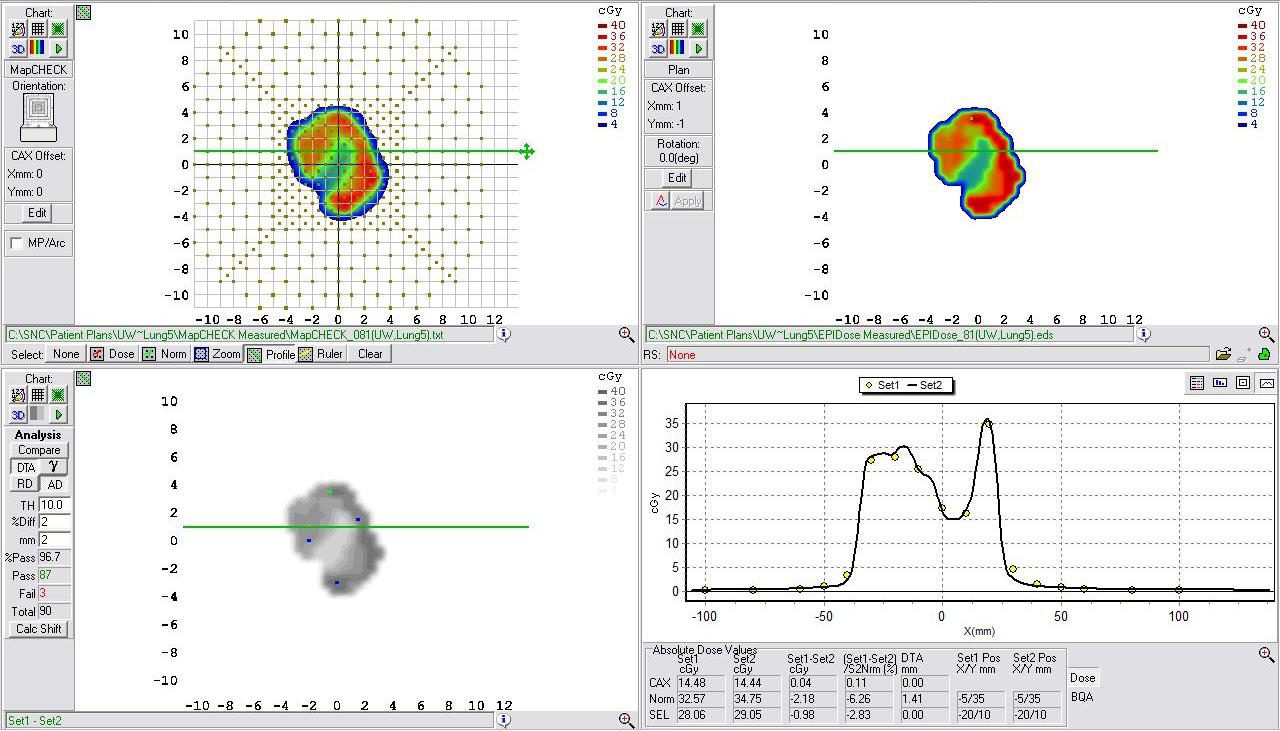
Example comparison of a converted EPID dose plane to a 2D diode array dose distribution for a typical lung field using a 2 mm/2% DTA comparison.

**Figure 9:**
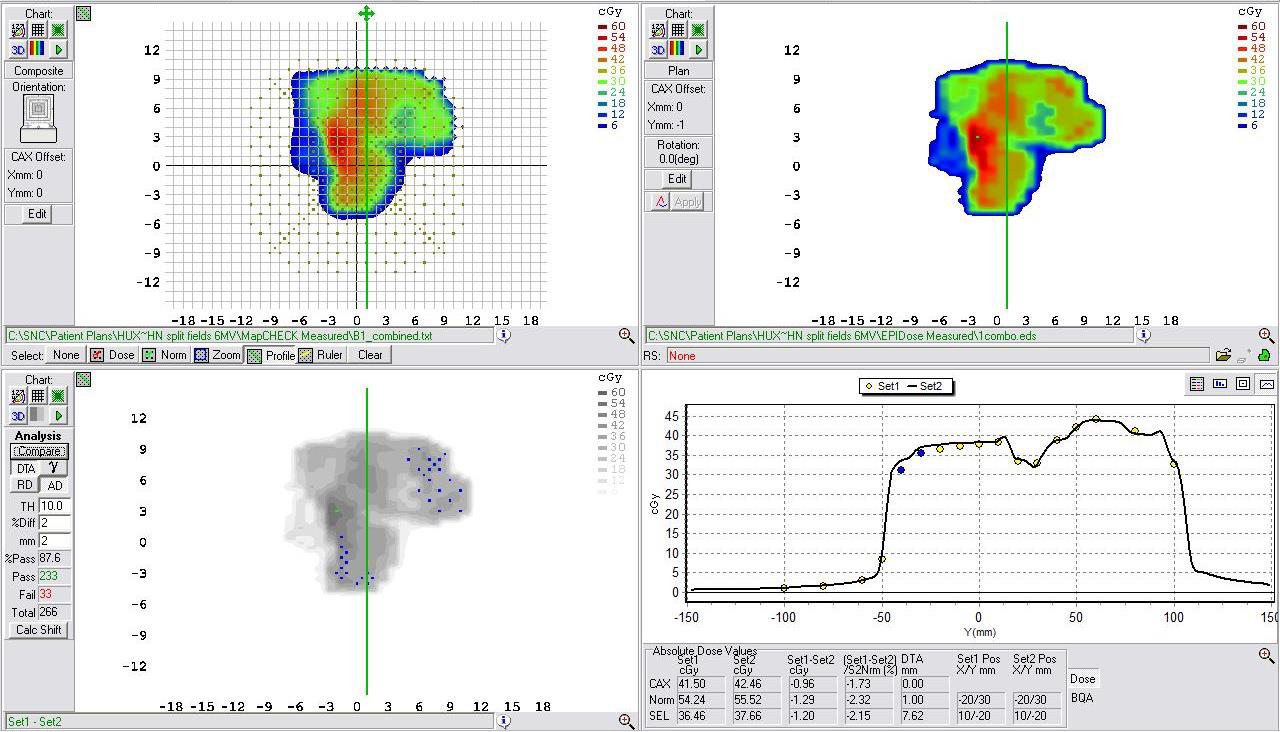
Example comparison of a converted EPID dose plane to a 2D diode array dose distribution for a typical head and neck field using a 2 mm/2% DTA comparison. This example illustrates a case in which lower pass rates, MapCHECK vs. EPIDose were found.

Subsets of the fields tested here were analyzed to determine if there was a significant effect of treatment site or beam energy. The most common site was prostate (n=142) whose fields had pass rates of 99.8%±0.1%, 98.3%±0.4%, and 86.4%±1.4%, for the three sets of comparison criteria used – 3 mm/3%, 2 mm/2%, and 1 mm/1%, respectively. For lung IMRT treatment fields (n=17), the pass rate was 99.7%±0.3%, 97.0%±1.2%, and 81.0%±4.1% and, for brain/head and neck fields (n=29), the pass rate was 99.2%±0.4%, 96.0%±1.2%, and 78.3%±2.6%. Figures 6–9 demonstrate representative 2 mm/2% comparison of sample fields from all treatment sites. The pass rate results show that the average pass rates decreases when the criteria are made more stringent, as would be expected.

A side note of some interest is that, for all treatment sites, the statistical 95% confidence intervals (around the pass rate means) associated with each criterion overlap over all treatment sites for the 3 mm/3% and 2 mm/2% pass rates, but not for the 1 mm/1% pass rate. This suggests that at the 1 mm/1% level of analysis, results begin to vary based on field size and complexity. In fact 1 mm/1% does not seem to be a clinically used criteria when doing planar dose comparisons.^(17)^ To summarize, our findings suggest that one can expect similar pass rates for different treatment sites in terms of the performance of the EPID IMRT QA system when compared to those measured with an IMRT QA 2D diode array.

Beams of different energies were considered separately in order to determine if there was a significant energy effect in terms of the performance of the EPID IMRT QA method. Individual EPIDose physics models were established for each distinct linac/energy combination. Fields with low energy (6 MV) were the most common (n=141) and had pass rates of 99.7%±0.1%, 97.8%±0.4%, 84.7%±1.3%, while high energy (10 or 15 MV) fields (n=47) had pass rates of 99.9%±0.1%, 98.0%±1.0%, and 84.5%±3.3% for 3 mm/3%, 2 mm/2%, and 1 mm/1%, respectively. Again, these findings suggest that one can expect similar pass rates regardless of the beam energy used in terms of the performance of the EPID IMRT QA system when compared to those measured with an IMRT QA 2D diode array.

**Figure 10:**
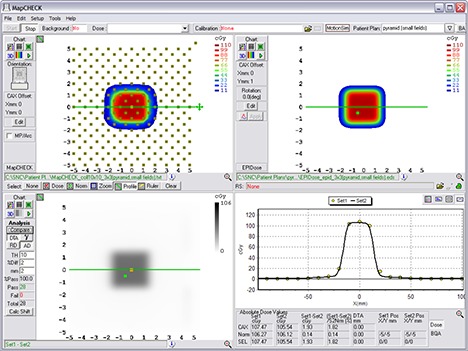
Example comparison of a converted EPID dose plane to a 2D diode array dose distribution for one of the nonclinical test fields analyzed – the 3×3 MLC field inside a 10×10 primary collimated area.

**Figure 11:**
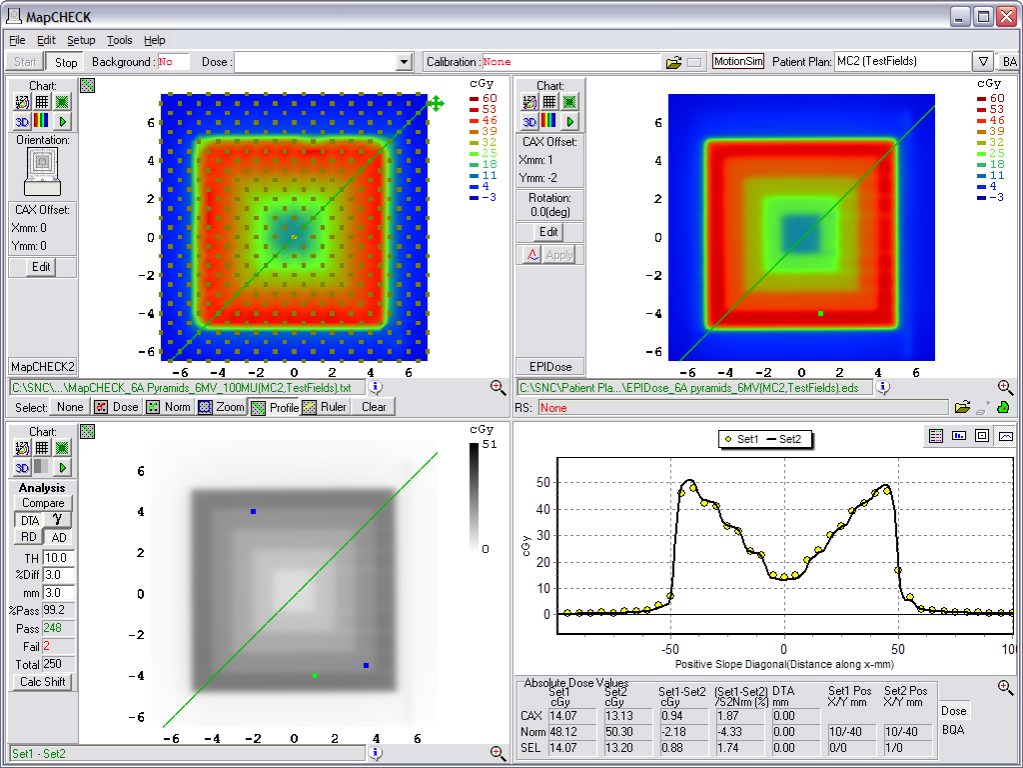
Example comparison of a converted EPID dose plane to a 2D diode array dose distribution for one of the nonclinical test fields analyzed – the 11‐segment, step‐and‐shoot inverted pyramid dose IMRT field.

For the nonclinical test fields, pass rates are shown in Table 4. Figures 10 and 11 show example planes and profiles from the 3×3 MLC/10×10 primary collimated field and the pyramid IMRT field, respectively.

**Table 4:** Percent difference/DTA comparison between EPID converted dose planes to 2D diode array measurements for seven designed test fields (Varian linac) and two analysis criteria (% difference/DTA) for 3 mm/3% and 2 mm/2% (DTA). The number of distinct diode measurement positions in the fields is shown, as some of these fields were quite small and did not cover many diodes in the MapCHECK array.

*Field Description*	*3 mm/3% Pass Rate*	*2 mm/2% Pass Rate*	*Points Analyzed*
1.0×1.0 MLC, 10×10 **Primary Jaws**	100.0	100.0	6
1.5×2.0 **MLC**, 10×10 **Primary Jaws**	100.0	91.7	12
2.0×1.0 **MLC**, 10×10 **Primary Jaws**	100.0	93.3	15
3.0×3.0 **MLC**, 10×10 **Primary Jaws**	100.0	100.0	28
5.0×5.0 **MLC**, 10×10 **Primary Jaws**	97.1	91.3	69
7.0×7.0 **MLC**, 10×10 **Primary Jaws**	94.2	88.3	121
**Inverted Dose Pyramid (11 MLC segments, fixed** 10×10 **Primary Jaws)**	99.2	88.4	250

Overall our results show that most of the fields measured with the commercial EPID conversion technique agree within 2 mm/2% of that measured with a currently used IMRT QA 2D diode array. The 2 mm/2% criterion is more stringent than that typically used for IMRT QA.^(14)^ ^,^ ^(17)^ This suggests that the EPID conversion technique discussed here may be acceptable for the purposes of routine clinical IMRT QA.

## IV. DISCUSSION

Our results demonstrate that the EPID dosimetry methodology described and analyzed in this work can be used to efficiently carry out the absolute dosimetry for IMRT treatment field quality assurance. We found that the described EPID method yields good agreement with measurements obtained from a 2D diode array, which has been widely employed for single field IMRT QA, for a number of common treatment sites and a number of photon energies that have been clinically employed for IMRT.

EPID dosimetry is still a relatively new field. While several methods of converting EPID images into a “dose” planes exist,^(5)^ ^,^ ^(18)^ some limitations stand out from previously used and described methods. Previously described methods of EPID IMRT QA either use software to estimate a “predicted” EPID image or predicted dose image (PDI), for comparison with the actual measured one^(5)^ ^,^ ^(7)^ or require that additional buildup to be placed directly on the EPID and require a film calibration.^(7)^ ^,^ ^(18)^

The strategy of using predicted portal images vs. measured images for IMRT QA,^(5)^ ^,^ ^(6)^ has the practical flaw that it does not fully audit the TPS dose algorithm, since a predicted image algorithm is used that is independent of the dose algorithm used for the generation of the actual treatment plan. Hence, errors in the TPS beam model (such as transmission, MLC penumbra, small/irregular segment head scatter corrections for dose output) may go undetected. Using PDI vs. measured EPID images may be useful in detecting machine performance errors, but are not useful in assessing the performance of the TPS dose algorithm and/or the treatment beam models used for treatment planning. IMRT QA errors often help a physicist improve the beam models present in the TPS and, therefore, using the TPS to calculate an IMRT dose per field (computing absolute dose planes in a phantom to compare against high density, high resolution measurements) is of vital importance for clinically relevant IMRT QA.

In the EPID method described and analyzed in this paper, all important conversion aspects are accounted for and, therefore, the converted EPID image can be compared directly with absolute planar dose. Back‐projecting the EPID image to the TPS source‐to‐dose plane distance (SPD) is an essential first step to account for beam divergence differences. Field size response differences are determined, as well. While it has been reported that an EPID output response can be matched to an output curve of absolute dose in phantom for a specific depth,^(7)^ this certainly was not entirely justified by the relative output curves shown in these studies, which varied much between institutions and certainly between EPID vendors. On the other hand, in order to compute the necessary correction to the input EPID image, the algorithm discussed in this study requires the MLC or DICOM RT Plan file, either of which contains the IMRT subsegments' weights, shapes, and locations for each field.

It has also been shown that corrections in the MLC transmission regions may be required to translate EPID exposure to dose equivalence.^(8)^ In the EPIDose algorithm analyzed in this study, this is achieved using the MLC file or DICOM RT Plan information and integrating small corrections based on transmission exposures.

The use of a dose redistribution kernel performs well (vs. an alternative of deconvolution into fluence first, then reestimation of dose in phantom) and is necessary because of the higher density material inherent to the EPID, which therefore yields a smaller dose kernel as compared to water. Hence, redistributing each of the EPID data points by a specific amount to the surrounding points stretches the EPID kernel into a water‐equivalent kernel. The model of the exact redistribution is treatment‐machine specific and EPID specific; however, similar models used for equivalent energies, desired QA depth equivalents, and EPID models appear equivalent.

Finally, the charge/dose response needs to be determined for the entire field to account for any variation in pixel responses. We have measured a linear response for EPID charge vs. delivered dose and no noticeable memory effect was found, which agrees with previously published results.^(19)^

The method of conversion described and analyzed in this study would not be appropriate for a composite treatment plan in which all treatment fields are delivered to a phantom at their actual treatment gantry angles, as each field delivered must be recorded as an individual image. EPIDose planes could, however, be summed for single gantry‐angle composite methods (all beams at nominal position treated to phantom), though this method is not necessarily encouraged by us, as it is less sensitive to both TPS and delivery errors compared to field‐by‐field analysis. This method would also not be appropriate for 4D IMRT QA, as the EPID is physically fixed relative to the treatment beam, with no method of reliably introducing motion to the detector.

**Figure 12:**
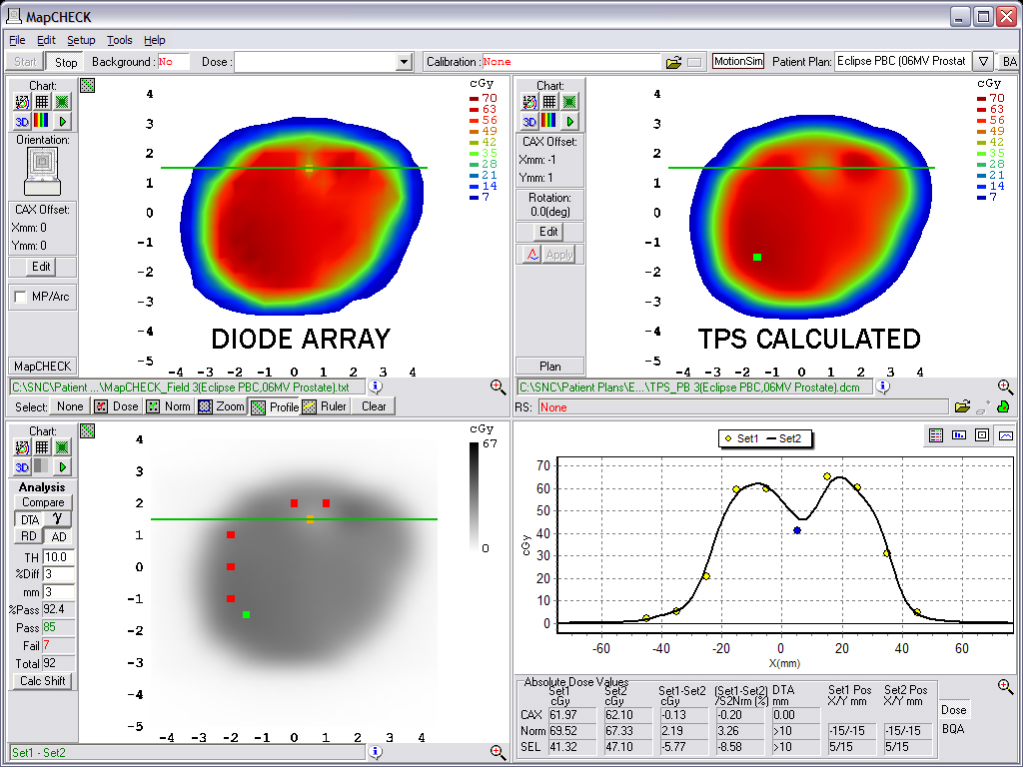
MapCHECK analysis of a TPS calculation that imperfectly models the high gradients of a DMLC IMRT field. Failing points are captured, but the lack of detector density does not make the source of error obvious.

**Figure 13:**
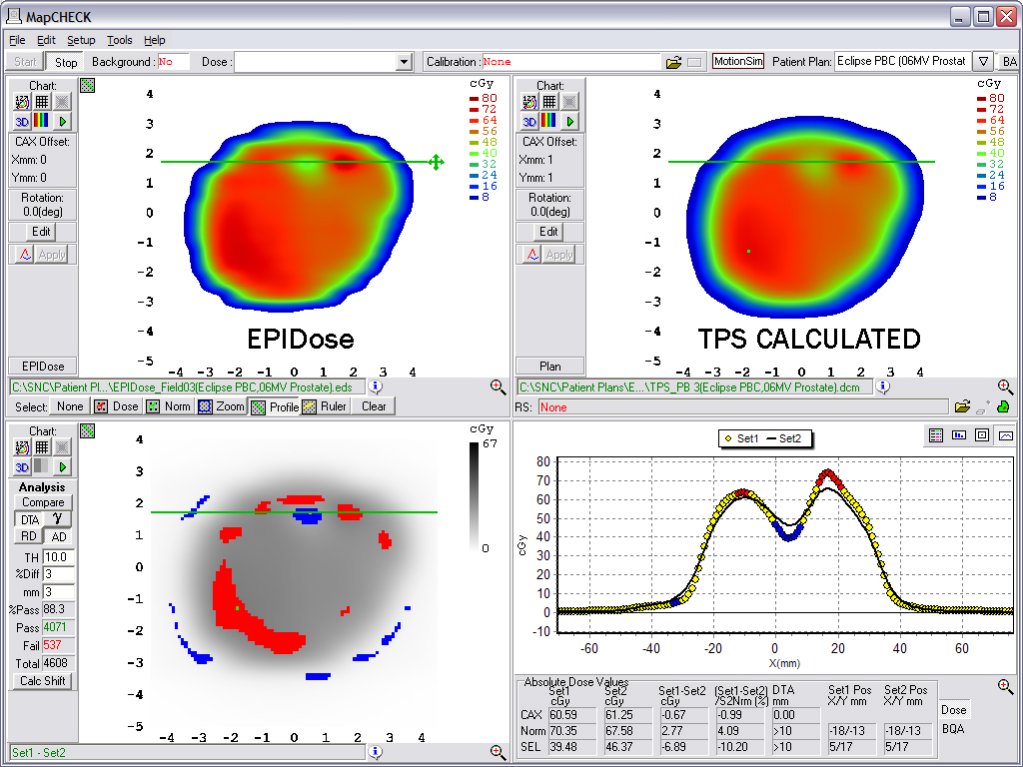
EPIDose analysis of the same DMLC IMRT field as in Fig.12. Here, failing regions are more obvious to the physicist, and the dose profiles lead to an easy diagnosis.

Finally, while common 2D arrays (diodes, ion chambers) are useful tools for detecting gross errors in delivery, the relatively low data density of these devices can make it difficult to determine very small regions of error such as tongue‐and‐groove effects. In addition, often large DTA tolerances are used in IMRT QA^(17)^ and these, combined with a noncontinuous density of discrete measurement points, can mask TPS modeling errors that result in imperfect calculations in high dose gradient regions. As an example, consider Figs. 12 and 13. Figure 12 illustrates an imperfect TPS beam model that does not model the IMRT gradients very well for a typical DMLC field. Here, the diode array analysis, though it captures failures along the gradients, does not make the errors abundantly obvious either in qualitative graphics or in pass rate. However, as Fig. 13 illustrates, the same field analyzed against EPIDose makes the error regions very obvious. Thus, the high density, high resolution method of EPID image conversion discussed in this paper, when appropriately modeled, may be able to determine effects such as these, and could be useful in commissioning new MLC and micro‐MLC systems, especially those designed for radiosurgery/small fields.

## V. CONCLUSIONS

Based on the strong agreement between EPIDose and MapCHECK point measurements, the EPIDose method of EPID image conversion to a dose plane as discussed in this study has all of the required characteristics for performing IMRT QA using a conventional method of per‐beam planar dose verification. The accuracy of the conversion process is independent of treatment site, as well as the treatment beam energy. The proposed method yields an accurate dose conversion with high data density, high efficiency, and a short calculation time.

## ACKNOWLEDGEMENTS

The authors would like to thank Ben Harris, (Kaiser Permenente, Portland, OR), John Duhon, (OncoLogics, Lafayette, LA), Bryan Coopey, (Allegheny General Hospital, Pittsburgh, PA), Shirley Yang, (Union Hospital/Hux Cancer Center, Terre Haute, IN), and Domenico Delli Carpini (Greenwich Hospital, Greenwich, CT) for their contribution of data to this project. Additionally, we would like to thank Sun Nuclear Corporation for the use of EPIDose and MapCHECK Software.

This work was partially supported by NIH NRSA 5 T32 CA009206 grant, NIH 1R01 CA109656, and by Canis Lupus LLC (Sauk County, WI).
